# Socioeconomic Factors and Inequality in the Prevalence and Treatment of Diabetes among Middle-Aged and Elderly Adults in China

**DOI:** 10.1155/2018/1471808

**Published:** 2018-12-27

**Authors:** Zhonghua Wang, Xiangjun Li, Mingsheng Chen

**Affiliations:** ^1^School of Health Policy & Management, Nanjing Medical University, 211166 Nanjing, China; ^2^Creative Health Policy Research Group, Nanjing Medical University, 211166 Nanjing, China; ^3^School of Health Economics and Management, Nanjing University of Chinese Medicine, 210023 Nanjing, China

## Abstract

**Background:**

In China, the prevalence of diabetes has increased significantly over recent decades, owing to the county's rapidly aging population. Although many studies have examined the prevalence of diabetes worldwide, there has been little analysis of the inequalities in its prevalence and treatment among middle-aged and elderly people.

**Objectives:**

This study evaluates influence factors and inequality in respect to the prevalence of diabetes and medication treatment among middle-aged and elderly Chinese adults.

**Methods:**

Data were obtained from the China Health and Retirement Longitudinal Study, a nationally representative household survey of middle-aged and elderly people (i.e., 45 years of age or older). Logistic regression models and the concentration index were used to estimate socioeconomic factors and inequalities in diabetes prevalence and treatment.

**Results:**

The prevalence of self-reported diabetes among middle-aged and elderly Chinese adults was 8.4%; this figure was significantly higher in urban areas than in rural areas. Concentrations of prevalence were observed among the poor in urban areas and among the rich in rural areas. Overall, the incidence of receiving antidiabetic medication among diabetes patients was 64.3%; this was significantly higher for individuals in urban areas than those in rural areas, suggesting that awareness of diabetes treatment in urban areas is better than that in rural areas. A disproportionate concentration of incidence of receiving antidiabetic medication was observed among the rich in both urban and rural areas. Socioeconomic factors significantly affected the prevalence of diabetes and the likelihood of receiving medication and are major contributors to inequality.

**Conclusion:**

In China, policies and strategies regarding diabetes prevention and control should further focus on associated socioeconomic factors and major contributors to reduce diabetes prevalence, improve diabetes treatment and management, and alleviate current inequality in the prevalence and treatment of diabetes among middle-aged and elderly adults.

## 1. Introduction

Diabetes is one of the fastest-growing chronic diseases in the world. In 2017, approximately 425 million adults worldwide had the condition, and about half of these cases had not been diagnosed. It is estimated that the number of people with diabetes will reach 629 million by 2045 [1]. According to the International Diabetes Federation's 2017 *Diabetes Atlas*, the prevalence of diabetes among women aged 20–79 years is about 8.4% globally; the corresponding rate among men is 9.1%, and approximately 4 million people aged 20–79 years died from diabetes in 2017, accounting for 10.7% of all-cause deaths worldwide [1]. Similar trends have been observed in China, where the prevalence of diabetes has increased markedly over recent decades [2]. According to the International Diabetes Federation's *Diabetes Atlas*, China currently has the largest number of people with diabetes in the world. In 2017, there were about 114.4 million people (aged between 20 and 79 years) with diabetes, and approximately 840,000 patients died of diabetes [1].

Diabetes is well known to be a chronic disease influenced by multiple factors. Although physiological or genetic factors play important parts in the condition, social and economic factors are the primary influence factors [3]. Many studies have reported that income, educational level, occupation, levels of physical activity, being overweight or obese, health behaviours, living conditions, and other demographic factors are strong influence factors of diabetes [4–6]. Socioeconomic position (SEP) indicators are particularly associated with the disease, with studies showing a link between diabetes prevalence and a disadvantaged SEP in both developed and developing countries [7–9]. Other studies have further found that people from high-income countries with a disadvantaged SEP were more likely to have diabetes, whereas the opposite association has been identified in people from low- and middle-income countries [10–12]. Several studies have suggested that a high level of education is associated with a low prevalence of diabetes [13–15], while the retired and people with “white collar” occupations have a higher prevalence of diabetes [16]. There may also be an association between socioeconomic factors and likelihood of receiving medication for diabetes. Some studies found a positive association between educational level and medication [17, 18]; however, others found the reverse [19]. Esmaeil-Nasab et al. [20] and Boutayeb et al. [21] reported that retired, unemployed, or single people were less likely to receive medication [20, 21]. Studies in China have found that being male, being single, living in a rural area, and having low SEP were associated with low levels of treatment [2, 22]. Disparities and inequalities may affect the prevalence of the disease, because its manifestation depends on the behaviour and lifestyle of individuals in all income groups [23]. Diabetes-related inequality is dynamic and dependent on cultural and societal development [24]. Moradi et al. [25] reported that diabetes inequality shifted from poor people to the rich in Iran, while other studies found an inverse relationship between socioeconomic inequality and the prevalence of diabetes in Germany and Portugal [26, 27]. Biswas et al. [28] revealed a disproportionate concentration of diabetes among the richest urban and the poorest rural populations in Bangladesh.

In recent years, the average age of the Chinese population has rapidly increased. Therefore, the prevalence of chronic diseases—diabetes in particular—has increased significantly [29]. Elderly people are more sensitive to healthcare utilization and the cost of chronic diseases, especially in rural areas, because of low incomes, absence of social security mechanisms, and poor healthcare awareness [30]. Many studies have examined the prevalence of diabetes and its influencing factors worldwide, but most have focused on the whole adult population rather than specific vulnerable groups therein. Furthermore, to our knowledge, no study yet has analysed the inequality in diabetes prevalence among middle-aged and elderly people in China or performed a comparative analysis of urban and rural areas from this perspective. Therefore, the aim of the present study is to analyse the influence factors and inequality in diabetes prevalence and treatment among middle-aged and elderly adults and to draw comparisons between urban and rural areas in China in this context as well.

## 2. Materials and Methods

### 2.1. Data Collection

The present study used data from the China Health and Retirement Longitudinal Study (CHARLS) 2015, which is available online at http://charls.ccer.edu.cn/en/page/data/2015-charls-wave1. This survey, which covered 150 counties in 28 provinces, constructed a high-quality, nationally representative sample of Chinese community-dwelling adults aged 45 years or older for scientific research [31]. The respondents to the survey included middle-aged and elderly adults in any household. The sampling involved four steps, as follows:

1. County-level units (counties or urban districts) were sampled directly; these counties cover 28 of 30 provinces in mainland China, excluding Tibet
2. Using recently updated village-level population data, village and community units within counties were chosen through reference to the National Bureau of Statistics. The administrative villages in rural areas and neighbourhoods in urban areas were used as primary sampling units (PSUs). Three PSUs were selected within each county, and a total of 450 PSUs were selected using the probability proportional to size sampling method
3. Household units were selected in each PSU, and the sampling frame was constructed using Google Earth base maps. GPS information was collected by photography at the door of the household. Then, the investigator interviewed the respondents and filled out each module of the questionnaire using a personal interview program on a portable computer. Data were uploaded to the project team over the internet at the end of the day
4. All age-eligible householders who were willing to participate in the survey were interviewed

The CHARLS collects detailed information about survey respondents and their spouses. The questionnaire includes information on basic demographics, two-week morbidity rates, chronic diseases, health status and behaviours, healthcare utilization and insurance, work, retirement and pension, and income, expenditures, and assets. Through the questionnaire, 14 chronic diseases were identified: hypertension, diabetes, dyslipidaemia, cancer/malignant tumour, chronic lung disease, liver disease, heart disease, stroke, kidney disease, stomach and other digestive diseases, emotional and psychiatric problems, memory-related disease, arthritis or rheumatism, and asthma. Diabetes was recorded when a respondent answered “yes” to either of the two questions “Have you been diagnosed with diabetes or high blood sugar by a doctor?” or “Do you take medicine or insulin injections to control your blood sugar?” and the question “When was the condition first diagnosed?”

The CHARLS did not collect any information on types of diabetes. We assumed that respondents who were diagnosed with diabetes before 19 years of age had type 1 diabetes, which is mainly caused by congenital genetic defects. Hence, middle-aged and elderly people who reported a diabetes diagnosis from before they turned 19 years old were excluded from this study. Respondents for whom there were any missing variables were also excluded, to ensure an accurate analysis. After the exclusion of these data, a total of 9739 participants were included in this analysis.

### 2.2. Data Analysis

Using this nationally representative survey data, we calculated the prevalence of self-reported diabetes and the incidence of receiving antidiabetic medication among middle-aged and elderly adults and estimated the effects of relevant socioeconomic factors thereon. Then, we used the concentration index (CI) and its decomposition to measure inequality in diabetes prevalence and treatment and to estimate the contributions of individual factors to this inequality. Statistical analyses were performed using Stata 14.0.

#### 2.2.1. Measuring Diabetes Prevalence and Treatment

The prevalence of self-reported diabetes was calculated among middle-aged and elderly adults enrolled in the survey in 2015 (*n* = 9739). Diabetes status was recorded by asking participants whether they had ever been diagnosed with diabetes by a physician. The prevalence of self-reported diabetes in rural and urban areas and in different income quintiles was calculated separately. The incidence of receiving antidiabetic medication was also calculated among middle-aged and elderly diabetes patients (*n* = 818). Antidiabetic medication was defined as oral antiglycaemic agents or insulin.

#### 2.2.2. Measuring Inequality in Diabetes Prevalence and Treatment

In order to reflect the inequality in diabetes prevalence, we used the concentration index to measure the distribution of the prevalence of self-reported diabetes and the incidence of receiving antidiabetic medication in relation to income, as follows [32]:

(1) $$\begin{array}{c} CI=\frac{2}{\mu }COV\left(h,r\right), \end{array}$$

where *r* is the fractional rank of individuals in the income distribution, *h* is whether or not an individual has diabetes, and *μ* is its mean. A positive concentration index value indicates a greater diabetes prevalence among the rich, while a negative value indicates that the poor are more likely to suffer from diabetes.

#### 2.2.3. Regression Analysis on Socioeconomic Factors and the Decomposition of the Concentration Index

In this study, we defined *X*_*k*_ as the socioeconomic factors related to diabetes prevalence. Thus, the linear regression analysis model of diabetes prevalence and related factors is as follows:

(2) $$\begin{array}{c} Y_{i}=\underset{k}{\sum }\beta_{k}X_{ki}+\varepsilon_{i}, \end{array}$$

where *Y* denotes the prevalence of self-reported diabetes or the incidence of receiving antidiabetic medication; *X*_*k*_ are related socioeconomic factors, including gender, age, ethnicity, marital status, education level, family size, household income per capita, work or type of labour undertaken, health status, and other health-related behaviours and lifestyle factors; and ε_*i*_ is the error term. Logistic regression modelling was used to estimate the factors related to the prevalence of diabetes, as *Y* is a binary variable.

The concentration index may be decomposed into the contributions of individual factors to diabetes prevalence inequality, in which each contribution is the product of the sensitivity of diabetes prevalence with respect to that factor and the degree of inequality in that factor. The concentration index decomposition was calculated as follows [33]:

(3) $$\begin{array}{c} CI=\underset{k}{\sum }\left(\frac{\beta_{k}}{{\bar{X}}_{k}}\right){CI}_{k}+\frac{G{CI}_{\varepsilon }}{\bar{Y}}, \end{array}$$

where *Y* is the mean of diabetes prevalence or the incidence of receiving antidiabetic medication; ${\bar{X}}_{k}$ is the mean of *X*_*k*_; CI_*k*_ is the concentration index for *X*_*k*_; and GCI_*ε*_ is the generalized concentration index for the error term *ε*.

## 3. Results

Detailed descriptions of the samples are provided in Table 1. Among the individuals included in the survey, 1921 were living in urban areas and 7818 were living in rural areas; 92.8% of respondents were of the Han ethnic group, 28.5% were older than 65 years of age, and 29.9% had hypertension.

The prevalence of self-reported diabetes by different income quintiles is presented in Table 2. As shown in the table, the prevalence of self-reported diabetes among middle-aged and elderly adults was 8.4%. Urban middle-aged and elderly adults demonstrated significantly higher prevalence (11.9%) than those in rural areas (6.8%). As seen in Table 2, too, there was also a statistically significant difference in diabetes prevalence by different income quintiles between urban and rural areas. Higher diabetes prevalence mainly appeared in the poorer and poorest quintiles in urban areas and in the richer and richest income quintiles in rural areas.

Table 3 presents the incidence of receiving antidiabetic medication in middle-aged and elderly diabetes patients. As shown, the overall incidence of receiving antidiabetic medication was 64.3%; this was significantly higher in urban patients (69.5%) than in rural individuals (61.9%). The highest incidence of receiving antidiabetic medication appeared in the richest income quintiles in both urban and rural areas.

Table 4 shows the regression results of the socioeconomic factors of self-reported diabetes prevalence among middle-aged and elderly adults. Gender was significantly associated with diabetes in both urban and rural areas: men were less likely to report diabetes in comparison with women (OR = 0.6247 and 0.6012). Elderly people older than 65 years were more likely to report diabetes when compared with individuals who were 55 or younger (OR = 1.3701 and 1.2282). Minority ethnic groups had a significantly lower likelihood of having diabetes than Han people; this effect was estimated to be greater in rural areas than in urban areas (OR = 0.4621 and 0.1128). Individuals who were married had a lower likelihood of having diabetes in both urban and rural areas (OR = 0.4955 and 0.6804), while those with a high level of education demonstrated a significantly lower likelihood of having diabetes in urban areas only (OR = 0.7955). As shown in Table 4, we found a significant association between household per capita income and diabetes prevalence, but this association exhibited a distinct difference between urban and rural areas. In urban areas, middle-aged and elderly adults with higher per capita incomes demonstrated a significantly lower likelihood of having diabetes (OR = 0.8997), while, in rural areas, the reverse was true (OR = 1.2604). In contrast with those who did less physical labour, the physical labour group exhibited a lower likelihood of having diabetes (OR = 0.8982 and 0.9268). Some health-related behaviours also significantly affected diabetes prevalence. Having never smoked significantly reduced an individual's risk of diabetes (OR = 0.5315 and 0.6520) while those who were overweight had a higher likelihood of having the disease (OR = 1.4370 and 1.7662). Middle-aged and elderly adults with hypertension had a dramatically higher likelihood of having diabetes; moreover, this effect was estimated to be greater in rural areas in comparison with urban areas (OR = 2.1442 and 2.9406).

Table 5 presents the results from the logistic regression model on the incidence of receiving antidiabetic medication among middle-aged and elderly patients. These results indicate significant correlations between socioeconomic factors and incidence of receiving antidiabetic medication. Urban individuals who were older than 55 years and had diabetes had a slightly lower likelihood of receiving antidiabetic medication (OR = 0.9817), while there was a significantly higher likelihood in rural areas (OR = 1.4237). Minority ethnic groups had a lower likelihood of receiving antidiabetic medication in comparison with the Han people in rural areas (OR = 0.4112), but there was no such significant correlation among urban individuals. Compared with divorced or single people, those who were married had a significantly higher likelihood of receiving antidiabetic medication in both urban and rural areas (OR = 1.2728 and 1.2486). Individuals with a high level of education had a higher likelihood of receiving antidiabetic medication; this effect was estimated to be greater in urban areas than in rural areas (OR = 1.3928 and 1.1749). There was a significant positive correlation between receiving antidiabetic medication and family size. Specifically, diabetes patients from large households were more likely to receive oral antiglycaemic agents or insulin (OR = 1.1804 and 1.4037). This effect was estimated to be more significant in rural areas. Household per capita income also positively affected the likelihood of receiving antidiabetic medication in both urban and rural areas (OR = 1.1682 and 1.4662). As shown in Table 5, the better an individual's self-reported health status, the lower their likelihood of receiving antidiabetic medication (OR = 0.6656 and 0.9338); this effect was estimated to be greater in urban areas.

Concentration index values for the prevalence of self-reported diabetes and receiving antidiabetic medication are reported in the last rows of Tables 2 and 3. As shown in Table 2, the concentration index of diabetes prevalence was positive overall (0.1217), indicating that the rich are at a higher risk of having diabetes. However, we found a great difference between urban and rural areas. The concentration index of diabetes prevalence was negative in urban areas (−0.1049), while it was positive in rural areas (0.1604). This indicates that diabetes is more frequent among the poor in urban areas, but not among the rich in rural areas. As shown in the last row of Table 3, the concentration index of the incidence of receiving antidiabetic medication was positive in both urban (0.1148) and rural (0.2041) areas. Rich middle-aged and elderly adults were more likely to receive antidiabetic medication, and the absolute value of the concentration index in rural areas was greater than that in urban areas.

Tables 6 and 7 show the contributions of each factor associated with inequality among middle-aged and elderly adults. Here, a positive contribution to inequality means that the relevant variable increases inequality, and vice versa. As shown in the fifth and the last columns of Table 6, the majority of the observed inequalities (−0.1049) in the prevalence of self-reported diabetes among urban middle-aged and elderly adults can be positively attributed to being older than 65 (30.31%), household per capita income (28.33%), having hypertension (26.35%), a body mass index (BMI) of between 18.5 and 24.1 (22.57%), and never having smoked (15.19%). Some other factors, such as being a farmer, marital status (unmarried), and gender (being female), had mostly negative contributions to the inequality. In rural areas, the major positive contribution to the inequality (0.1604) was related to being older than 65 (37.75%), household per capita income (35.23%), obesity (BMI > 24.1) (27.24%), being a member of an ethnic minority group (21.68%), and having hypertension (17.17%). Never smoking and self-reported health status had mostly negative contributions. As shown in the fifth and the last columns of Table 7, the main positive contributions to the inequality in incidence of receiving antidiabetic medication among urban middle-aged and elderly diabetes patients were household per capita income (29.79%), self-reported health status (19.09%), upper secondary and vocational training (17.64%), being married (13.84%), and age (i.e., being older than 65) (12.01%). In rural areas, the main positive contributors were household per capita income (35.39%), secondary and vocational training (28.27%), family size (21.84%), and ethnic minority status (19.18%). Family size had the main negative contribution to the inequality in urban areas.

## 4. Discussion

In this study, 8.4% of the respondents reported diabetes and 64.3% of middle-aged and elderly diabetes patients were receiving treatment. Some socioeconomic factors, such as gender, age, ethnicity, marital status, educational level, per capita incomes, and physical conditions, significantly affected the prevalence of self-reported diabetes and the likelihood of receiving medication. Our study also revealed that, although there was a disproportionate concentration of diabetes prevalence among rich middle-aged and elderly adults overall, it was more concentrated in low-income groups in urban areas but high-income groups in rural areas. In addition, we found a disproportionate concentration of individuals receiving antidiabetic medication among the rich in both urban and rural areas. Socioeconomic factors such as being older than 65 years, household per capita income, hypertension, self-reported health status, level of education, family size, and BMI were major contributors to the inequality in diabetes prevalence and receiving antidiabetic medication among middle-aged and elderly adults.

As noted, 8.4% of the study's respondents reported diabetes, which is a lower figure than that reported in the International Diabetes Federation's *Diabetes Atlas* (10.9%) [1]. We also found that the prevalence of self-reported diabetes was significantly higher in urban areas than in rural areas, consistent with some previous studies [2, 6, 9, 14]. This finding could be attributed to the substantial difference in economic development between urban and rural areas in China [34]. In general, urban residents may have higher incomes and higher carbohydrate intake, but do less physical labour, which may ultimately produce a relatively high prevalence of diabetes. Middle-aged and elderly women have a significantly greater likelihood of diabetes than men according to our results which is again in agreement with the previous studies [11, 12, 15]; this result may be related to the menopause. Older people were found to have a higher prevalence of diabetes, as reported by most previous studies in this field [3, 5, 11–15, 27, 35]. Marriage is protective of health in general, and this is especially true among older couples [36]. Our analysis also showed that married people were less likely to have diabetes, in both urban and rural areas in China. The Han people had a significantly higher likelihood of having diabetes than other ethnic groups, especially in rural China. This is consistent with the results of the Survey of Chronic Diseases Surveillance in China [37]. Our study also found that middle-aged and elderly adults with high levels of education were less likely to have diabetes, but this effect was not significant in rural areas. In addition, individuals who are engaged in physical occupations have a lower likelihood of having diabetes than those who are not; this finding is similar to those of the previous studies [6, 14, 38]. Therefore, some initiatives could be taken to reduce the risk of developing diabetes in urban individuals with lower education levels and those engaged in less physical occupations, such as officials, salespersons, and other white-collar occupations.

Furthermore, we found that a high prevalence of self-reported diabetes mainly appeared in relatively low-income quintiles in urban areas and high-income quintiles in rural areas (Table 2). The regression results of Table 4 further demonstrate that there is a negative association between household per capita income and diabetes prevalence in urban areas but a positive one in rural areas. This finding reflects the huge gap in socioeconomic structure and development between urban and rural areas in China. The possible explanation for this discrepancy may be that there is a threshold of income, living conditions, or development status in the process of social and economic development, above which the likelihood of having diabetes decreases with increasing per capita income, but below which the reverse is true. The finding that some health-related factors, such as smoking and being overweight, significantly increased the prevalence of self-reported diabetes is in agreement with the literature [4, 11, 14]. Notably, hypertension was associated with a dramatically higher likelihood of diabetes, especially in rural areas. Hence, improved strategies for the prevention and control of hypertension may be important in terms of reducing the risk of diabetes.

Although a previous study had found that 25.8% of Chinese adults with diabetes were receiving treatment [2], the treatment rate among middle-aged and elderly diabetes patients in our study was comparatively high (64.3%). Individuals in urban areas were significantly more likely to receive medication than those in rural areas (Table 3), indicating that awareness of diabetes treatment is better in urban areas than in rural ones. Marriage and family life were also positive factors, making it more likely for middle-aged and elderly adults with diabetes to receive antidiabetic medication. Education had a stronger positive influence on the treatment of diabetes, similar to that found by previous studies [17, 18, 35]; a higher educational level is an indicator of the ability to translate information into practical behaviours and thus to regularly manage and control chronic diseases [11]. Diabetes patients with large families were more likely to receive medication treatment; this was more common in rural areas. This finding reflects that a large family, especially in rural areas, is strongly supportive of elderly diabetes patients both emotionally and financially in the treatment and management of their chronic diseases. In addition, we found that the increase in household per capita income significantly enhanced the likelihood of receiving antidiabetic medication (Table 5). This finding indicates that the economic factor plays an important role in the medication treatment of diabetes; strategies for increasing income may be a practicable way to improve the ability and awareness of diabetes treatment among middle-aged and elderly adults.

This study found a significant difference in the inequality of the prevalence of self-reported diabetes between urban and rural areas. Owing to the dramatic rural-urban income gap, there are differences in lifestyle and awareness of the disease: urban high-income groups are better educated and have a healthier lifestyle than low-income groups. However, in rural areas, individuals in the relatively high-income groups may have only just emerged from poverty and, while still lacking health consciousness and health-promoting behaviours, develop unhealthy lifestyle habits such as a high-fat and high-calorie diet [22]. Therefore, diabetes is more concentrated in low-income groups in urban areas but high-income groups in rural areas. Furthermore, we observed that factors such as age (i.e., being more than 65 years old), household per capita income, hypertension, and BMI were major contributors to the inequality in diabetes prevalence among middle-aged and elderly adults. Policies aimed at reducing inequality in diabetes prevalence should focus on these socioeconomic factors, for example, by implementing strategies that strengthen health education and nutritional intervention, control and prevent hypertension and obesity, and narrow the income gap. The disproportionate concentration of receiving antidiabetic medication among the rich indicates that high-income groups have a greater ability to treat and manage their disease in comparison with low-income groups among middle-aged and elderly adults. Factors such as household per capita income, self-reported health status, level of education, and family size were all major contributors to the inequality. Policy efforts should additionally focus on improving the education levels of the poor in both urban and rural areas, adjusting population strategies to exert the protective effect of family size in rural areas, and narrowing the income gap to reduce inequality of treatment among middle-aged and elderly adults.

### 4.1. Strength and Limitations of the Study

The China Health and Retirement Longitudinal Study is a high-quality, nationally representative sample of middle-aged and elderly individuals. The findings of this study provide important evidence on the influence of social and economic factors on the prevalence and management of diabetes among middle-aged and elderly people. However, some limitations of our study must be acknowledged. Since the CHARLS did not include any information on fasting plasma glucose, the diabetes prevalence figures we used were obtained only by asking elderly adults whether they had ever been diagnosed with diabetes by a physician and did not take into account those who might have had diabetes but were not or did not recall being diagnosed. Thus, the true diabetes prevalence might have been underestimated to some extent. In addition, our study findings are likely to have been influenced by additional factors that were not included in the CHARLS.

### 4.2. Implications to Practice and Research

Policies aimed at reducing diabetes prevalence and inequality in China should further focus on the socioeconomic factors influencing chronic diseases among middle-aged and elderly adults. Appropriate strategies with regard to population, income distribution, and education promotion could be implemented to reduce inequality and improve diabetes treatment among middle-aged and elderly adults. Future research should be directed towards effective interventions to reduce diabetes prevalence and inequality and to improve diabetes management among middle-aged and elderly adults in China.

## 5. Conclusions

Chronic diseases occur more frequently among middle-aged and elderly people. Our findings reveal a prevalence of self-reported diabetes among Chinese middle-aged and elderly people of 8.4% and a higher prevalence in urban areas than in rural areas. Although the rich have a higher risk of diabetes overall, the condition is observed more frequently among the poor in urban areas and the rich in rural areas; 64.3% of middle-aged and elderly diabetes patients were receiving treatment, and a disproportionate concentration of individuals receiving treatment among the rich was observed in both urban and rural areas. Some socioeconomic factors significantly affect diabetes prevalence and the likelihood of receiving treatment and are major contributors to their inequalities.

## Figures and Tables

**Table 1:** Description of samples.

	Urban areas		Rural areas		Total	
	*n*	%	*n*	%	*n*	%
Gender						
Male	995	51.80%	3581	45.80%	4576	46.99%
Female	926	48.20%	4237	54.20%	5163	53.01%
Age group (years)						
45–55	537	27.95%	2611	33.40%	3148	32.32%
56–65	728	37.90%	3087	39.49%	3815	39.17%
>65	656	34.15%	2120	27.12%	2776	28.50%
Ethnicity						
Han	1794	93.39%	7247	92.70%	9041	92.83%
Other ethnic groups	127	6.61%	571	7.30%	698	7.17%
Marital status						
Divorced/single/separated	259	13.48%	996	12.74%	1255	12.89%
Married/partnered	1662	86.52%	6822	87.26%	8484	87.11%
Education level						
Less than primary school	387	20.15%	4089	52.30%	4476	45.96%
Less than lower secondary	933	48.57%	3280	41.95%	4213	43.26%
Upper secondary, vocational training	601	31.29%	449	5.74%	1050	10.78%
Family size						
≤2	801	41.70%	2733	34.96%	3534	36.29%
≥3	1120	58.30%	5085	65.04%	6205	63.71%
Household income per capita (mean + SD)	17,369	957.04	6611	400.23	11,019	555.23
Type of labour						
Farmer	589	30.66%	6324	80.89%	6913	70.98%
Self-employed/employed	975	50.75%	1352	17.29%	2327	23.89%
Government & institutions	357	18.58%	142	1.82%	499	5.12%
Drinking						
Never drunk	960	49.97%	4467	57.14%	5427	55.72%
History of drinking	961	50.03%	3351	42.86%	4312	44.28%
Smoking						
Never smoked	1095	57.00%	4509	57.67%	5604	57.54%
History of smoking	826	43.00%	3309	42.33%	4135	42.46%
ADL						
Without ADL	1779	92.61%	7018	89.77%	8797	90.33%
With any ADL	142	7.39%	800	10.23%	942	9.67%
BMI						
≤18.5	801	41.70%	3992	51.06%	4793	49.21%
18.6–24.1	70	3.64%	513	6.56%	583	5.99%
>24.1	1050	54.66%	3313	42.38%	4363	44.80%
Self-reported health (mean + SD)	3.7315	0.0267	3.9175	0.0201	3.8413	0.0174
Has hypertension						
No	1246	64.86%	5581	71.39%	6827	70.10%
Yes	675	35.14%	2237	28.61%	2912	29.90%

Note: calculations were weighted using individual sampling weights and adjusted for household and individual responses.

**Table 2:** Prevalence of self-reported diabetes and concentration index among middle-aged and elderly adults.

	Total		Urban areas		Rural areas		*p* value
	Mean	SD	Mean	SD	Mean	SD	
Poorest	0.0493	0.0058	0.1389	0.0154	0.0372	0.0056	.014
Poorer	0.0640	0.0069	0.1293	0.0203	0.0568	0.0070	.048
Middle	0.0868	0.0070	0.1096	0.0425	0.0493	0.0073	.024
Richer	0.0873	0.0073	0.0825	0.0188	0.0892	0.0063	.000
Richest	0.0890	0.0096	0.0928	0.0332	0.0827	0.0077	.000
Total	0.0844	0.0037	0.1185	0.0103	0.0677	0.0033	.000
Concentration index	0.1217		−0.1049		0.1604		

Notes: *n* = 9739. Calculations were weighted using individual sampling weights and adjusted for household and individual responses.

**Table 3:** Incidence of receiving antidiabetic medication and concentration index among middle-aged and elderly diabetes patients.

	Total		Urban areas		Rural areas		*p* value
	Mean	SD	Mean	SD	Mean	SD	
Poorest	0.5062	0.0513	0.6499	0.1143	0.4816	0.0564	.008
Poorer	0.5211	0.0432	0.6391	0.157	0.5017	0.0448	.045
Middle	0.6812	0.0434	0.7603	0.0761	0.6630	0.0509	.002
Richer	0.6769	0.0590	0.7748	0.1016	0.6589	0.0644	.055
Richest	0.7495	0.0393	0.7993	0.044	0.6872	0.0801	.019
Total	0.6425	0.0217	0.6952	0.0337	0.6199	0.0253	.000
Concentration index	0.2483		0.1148		0.2041		

Notes: *n* = 818. Calculations were weighted using individual sampling weights and adjusted for household and individual responses.

**Table 4:** Logistic regression model on prevalence of self-reported diabetes among middle-aged and elderly adults.

	Urban areas		Rural areas	
	Odds ratio	95% CI	Odds ratio	95% CI
Gender (ref. female)	0.6247^∗∗∗^	(0.4451-0.8768)	0.6012^∗∗∗^	(0.4606-0.7847)
Age (ref. ≤ 55)				
56–65	1.0587	(0.6690-1.3737)	1.1315	(0.9017-1.4200)
>65	1.3701^∗∗^	(0.9651-1.5449)	1.2282^∗∗^	(0.9756-1.3944)
Ethnicity (ref. Han)	0.4621^∗∗^	(0.2451-0.8714)	0.1128^∗∗∗^	(0.0608-0.2094)
Marital status (ref. divorced/single)	0.4955^∗∗∗^	(0.3428-0.7162)	0.6804^∗∗∗^	(0.5214-0.8878)
Education level (ref. less than primary school)				
Less than lower secondary	1.0262	(0.7194-1.4637)	0.9310	(0.7556-1.1471)
Upper secondary, vocational training	0.7955^∗^	(0.5206-1.1156)	1.0427	(0.6780-1.6034)
Family size (ref. ≤ 2)	0.7490^∗∗^	(0.5656-0.9918)	0.9001	(0.7397-1.0952)
Household income per capita (log)	0.8997^∗^	(0.8325-1.1723)	1.2604^∗∗^	(1.1228-1.6047)
Type of labour (ref. government & institutions)				
Farmer	0.8982^∗^	(0.7285-1.1934)	0.9268^∗∗^	(0.7133-1.1941)
Self-employed/employed	1.4465	(0.9669-2.1637)	0.9015^∗^	(0.5274-1.7012)
Never drunk (ref. history of drinking)	0.9486	(0.6996-1.6862)	0.8158^∗^	(0.6534-1.0186)
Never smoked (ref. history of smoking)	0.5315^∗∗∗^	(0.3749-0.7535)	0.6520^∗∗∗^	(0.5019-0.8471)
ADL (ref. without any ADL)	1.2659	(0.7874-2.0351)	1.5412^∗∗∗^	(1.1809-2.0115)
BMI (ref. ≤ 18.5)				
18.6–24.1	0.0753^∗∗^	(0.0109-0.5184)	0.5628^∗^	(0.3089-1.0251)
>24.1	1.4370^∗∗^	(1.1749-1.8209)	1.7662^∗∗∗^	(1.4432-2.1615)
Self-reported health status	0.7683^∗∗^	(0.4993-0.9937)	0.9706^∗∗∗^	(0.8049-1.2060)
Having hypertension (ref. no)	2.1442^∗∗∗^	(1.6140-2.8485)	2.9406^∗∗∗^	(2.4204-3.5724)

Note: estimates were weighted using individual sampling weights and adjusted for household and individual responses. ^∗∗∗^*p* < .001, ^∗∗^*p* < .01, and ^∗^*p* < .05.

**Table 5:** Logistic regression model on incidence of receiving antidiabetic medication among middle-aged and elderly diabetes patients.

	Urban		Rural	
	Odds ratio	95% CI	Odds ratio	95% CI
Gender (ref. female)	0.5551	(0.2141-1.4391)	0.9052	(0.5207-1.5739)
Age (ref. ≤ 55)				
56–65	0.9817^∗∗^	(0.6663-1.3928)	1.4237^∗^	(0.9608-2.1761)
>65	0.8092^∗^	(0.4745-1.2771)	1.0788	(0.5417-1.9686)
Ethnic group (ref. Han)	0.7072	(0.2122-2.3567)	0.4112^∗∗^	(0.1715-0.8863)
Marital status (ref. divorced/single)	1.2728^∗∗^	(0.8341-1.6024)	1.2486^∗∗∗^	(0.7657-1.5948)
Education level (ref. less than primary school)				
Less than lower secondary	1.0340	(0.3987-2.9754)	1.1415	(0.7232-1.9017)
Upper secondary & vocational training	1.3928^∗∗∗^	(0.9033-1.6541)	1.1749^∗∗^	(0.5098-1.7854)
Family size (ref. ≤ 2)	1.1804^∗^	(0.5426-1.8329)	1.4037^∗∗^	(0.8316-1.8021)
Household income per capita (log)	1.1682^∗∗^	(0.6530-1.6392)	1.4662^∗∗∗^	(0.7751-1.7998)
Type of labour (ref. government & institutions)				
Farmer	0.7636	(0.2988-2.1452)	0.8289	(0.4446-1.9450)
Self-employed/employed	2.6591^∗∗^	(1.0008-4.0149)	3.4054	(0.3585-9.3468)
Never drunk (ref. history of drinking)	0.9704	(0.4338-2.1709)	1.3116	(0.8168-2.3085)
Never smoked (ref. history of smoking)	0.9883	(0.3788-2.5791)	0.9144	(0.5186-2.6123)
ADL (ref. without any ADL)	2.3800	(0.6907-8.2009)	1.3776	(0.7673-2.4743)
BMI (ref. ≤ 18.5)				
18.6–24.1	1.0009	(0.2321-2.4933)	1.1721	(0.4499-3.0530)
>24.1	0.8983	(0.3890-2.0746)	1.2010	(0.7845-1.8385)
Self-reported health status	0.6656^∗∗∗^	(0.2617-0.9981)	0.9338^∗^	(0.5725-1.5652)
Having hypertension (ref. no)	1.6678^∗^	(0.7710-2.4429)	0.9856	(0.4700-2.0499)

Note: estimates were weighted using individual sampling weights and adjusted for household and individual responses. ^∗∗∗^*p* < .001, ^∗∗^*p* < .01, ^∗^*p* < .05.

**Table 6:** Decomposition of concentration index of self-reported diabetes prevalence among middle-aged and elderly adults.

	Urban				Rural			
	Elasticity	Concentration index	Contribution	Percentage contribution (%)	Elasticity	Concentration index	Contribution	Percentage contribution (%)
Gender (ref. female)	−6.5950	−0.0019	0.0125	−11.95	−7.8291	0.0013	−0.0096	−5.97
Age (ref. ≤ 55)								
56–65	0.1442	−0.0508	−0.0073	6.98	0.2118	−0.0834	−0.0177	−11.01
>65	0.6961	−0.0457	−0.0318	30.31	1.0458	0.0579	0.0606	37.75
Ethnic group (ref. Han)	−1.7174	0.0081	−0.0139	13.26	−3.7391	−0.0093	0.0348	21.68
Marital status (ref. divorced/single)	−2.6160	−0.0072	0.0188	−17.94	−1.8089	−0.0063	0.0114	7.10
Education level (ref. less than primary school)								
Less than lower secondary	0.1140	−0.0124	−0.0014	1.35	−0.5347	−0.0047	0.0025	1.57
Upper secondary & vocational training	−0.1217	0.0214	−0.0026	2.48	0.0442	0.3213	0.0142	8.85
Family size (ref. ≤ 2)	−1.4028	−0.0069	0.0098	−9.37	−1.2574	0.0057	−0.0072	−4.47
Household income per capita (log)	−6.4597	0.0046	−0.0297	28.33	7.5399	0.0075	0.0565	35.23
Type of labour (ref. government & institutions)								
Farmer	−0.5484	−0.0417	0.0229	−21.83	−0.2877	0.0098	−0.0028	−1.76
Self-employed/employed	0.6936	0.0027	0.0019	−1.79	−0.4344	0.0007	−0.0003	−0.19
Never drunk (ref. history of drinking)	−0.2423	0.0491	−0.0119	11.34	−1.1329	0.0080	−0.0091	−5.65
Never smoked (ref. history of smoking)	−1.0816	0.0147	−0.0159	15.19	−3.2010	0.0101	−0.0323	−20.16
ADL (ref. without any ADL)	0.1964	−0.0443	−0.0087	8.29	0.3602	−0.0139	−0.0050	−3.12
BMI (ref. ≤ 18.5)								
18.6–24.1	−0.8901	0.0266	−0.0237	22.57	−0.6723	−0.0195	0.0131	8.17
>24.1	1.8871	−0.0075	−0.0142	13.49	3.2644	0.0134	0.0437	27.24
Self-reported health status	−4.1105	−0.0022	0.0090	−8.62	−2.8108	0.0059	−0.0166	−10.34
Having hypertension (ref. no.)	2.6581	−0.0104	−0.0276	26.35	5.3988	0.0051	0.0275	17.17
Total				108.44				102.09

**Table 7:** Decomposition of concentration index of incidence of receiving antidiabetic medication among middle-aged and elderly diabetes patients.

	Urban				Rural			
	Elasticity	Concentration index	Contribution	Percentage contribution (%)	Elasticity	Concentration index	Contribution	Percentage contribution (%)
Gender (ref. female)	−1.5522	0.0039	−0.0061	−5.27	−0.2610	0.0145	−0.0038	−1.85
Age (ref. ≤ 55)								
56–65	−0.0895	−0.0168	0.0015	1.31	0.2074	−0.0434	−0.0090	−4.46
>65	−0.2097	−0.0657	0.0138	12.01	0.0559	0.1625	0.0091	4.45
Ethnic group (ref. Han)	−0.2837	−0.0181	0.0051	4.47	−1.4342	−0.0273	0.0392	19.18
Marital status (ref. divorced/single)	0.6566	0.0242	0.0159	13.84	0.5534	0.0173	0.0118	8.49.
Education level (ref. less than primary school)								
Less than lower secondary	0.0158	−0.0324	−0.0005	−0.45	0.0762	−0.0747	−0.0057	−2.79
Upper secondary & vocational training	0.1479	0.1369	0.0202	17.64	0.1107	0.5213	0.0577	28.27
Family size (ref. ≤ 2)	0.1256	−0.1058	−0.0133	−11.58	0.2597	0.1717	0.0446	21.84
Household income per capita (log)	0.5740	0.0596	0.0342	29.79	0.9478	0.0762	0.0722	35.39
Type of labour (ref. government & institutions)								
Farmer	−0.3084	−0.0317	0.0098	8.52	−0.1197	0.0849	−0.0102	−4.98
Self-employed/employed	0.1532	0.0227	0.0035	3.03	0.4438	0.0031	0.0014	0.67
Never drunk (ref. history of drinking)	−0.2135	0.0098	−0.0021	−1.82	0.4319	0.0110	0.0048	2.33
Never smoked (ref. history of smoking)	−0.1346	0.0027	−0.0004	−0.35	−0.1928	0.0120	−0.0023	−1.13
ADL (ref. without any ADL)	0.1245	−0.0213	−0.0027	−2.31	0.0582	−0.1189	−0.0069	−3.39
BMI (ref. ≤ 18.5)								
18.6–24.1	0.0011	0.1166	0.0001	0.11	0.0474	−0.0905	−0.0043	−2.10
>24.1	−0.0966	−0.0805	0.0078	6.77	0.2291	0.0974	0.0223	10.93
Self-reported health status	−1.9222	−0.0114	0.0219	19.09	−0.7048	−0.0149	0.0105	5.15
Having hypertension (ref. no)	0.0675	−0.0264	−0.0018	−1.55	−0.0125	0.0255	−0.0003	−0.16
Total				93.25	107.35			

## Data Availability

The data used to support the findings of this study are available from the corresponding author upon request.
